# Appropriateness of Initial Course of Action in the Management of Blunt Trauma Based on a Diagnostic Workup Including an Extended Ultrasonography Scan

**DOI:** 10.1001/jamanetworkopen.2022.45432

**Published:** 2022-12-07

**Authors:** Fanny Planquart, Emmanuel Marcaggi, Raiko Blondonnet, Olivier Clovet, Xavier Bobbia, Bastien Boussat, Julien Pottecher, Tobias Gauss, Laurent Zieleskiewicz, Pierre Bouzat

**Affiliations:** 1 Service d’Anesthésie-Réanimation et Médecine Péri-Opératoire, Hôpitaux Universitaires de Strasbourg, Hôpital de Hautepierre, Strasbourg, France; 2 Pôle d’Anesthésie-Réanimation, CHU Grenoble Alpes, Grenoble, France; 3 Pôle de Médecine Périopératoire, CHU Clermont-Ferrand, Clermont-Ferrand, France; 4 Département d’Anesthésie-Réanimation et Médecine Péri-Opératoire, Groupe Hospitalier Pitié-Salpêtrière, Assistance Publique Hôpitaux de Paris, Paris, France; 5 Université de Montpellier, Département Urgences CHU Montpellier, Montpellier, France; 6 Service d’épidémiologie et évaluation médicale, CHU Grenoble-Alpes, laboratoire TIMC-IMAG, UMR 5525 Joint Research Unit, Centre National de Recherche Scientifique, Université Grenoble-Alpes, France; 7 Université de Strasbourg, Fédération de Médecine Translationnelle de Strasbourg, UR3072, Strasbourg, France; 8 Service d’anesthésie réanimation, Hôpital Nord, Assistance Publique-Hôpitaux de Marseille, Marseille, Centre de recherche en Cardiovasculaire et Nutrition, Aix-Marseille Université, France; 9 University Grenoble Alpes, Inserm, U1216, CHU Grenoble Alpes, Grenoble Institut Neurosciences, Grenoble, France

## Abstract

**Importance:**

The extended Focused Assessment With Sonography for Trauma (E-FAST) has become a cornerstone of the diagnostic workup in patients with trauma. The added value of a diagnostic workup including an E-FAST to support decision-making remains unknown.

**Objective:**

To determine how often an immediate course of action adopted in the resuscitation room based on a diagnostic workup that included an E-FAST and before whole-body computed tomography scanning (WBCT) in patients with blunt trauma was appropriate.

**Design, Setting, and Participants:**

This cohort study was conducted at 6 French level I trauma centers between November 5, 2018, and November 5, 2019. Consecutive patients treated for blunt trauma were assessed at the participating centers. Data analysis took place in February 2022.

**Exposures:**

Diagnostic workup associating E-FAST (including abdominal, thoracic, pubic, and transcranial Doppler ultrasonography scan), systematic clinical examination, and chest and pelvic radiographs.

**Main Outcomes and Measures:**

The main outcome criterion was the appropriateness of the observed course of action (including abstention) in the resuscitation room according to evaluation by a masked expert panel.

**Results:**

Of 515 patients screened, 510 patients (99.0%) were included. Among the 510 patients included, 394 were men (77.3%), the median (IQR) age was 46 years (29-61 years), and the median (IQR) Injury Severity Score (ISS) was 24 (17-34). Based on the initial diagnostic workup, no immediate therapeutic action was deemed necessary in 233 cases (45.7%). Conversely, the following immediate therapeutic actions were initiated before WBCT: 6 emergency laparotomies (1.2%), 2 pelvic angioembolisations (0.4%), 52 pelvic binders (10.2%), 41 chest drains (8.0%) and 16 chest decompressions (3.1%), 60 osmotherapies (11.8%), and 6 thoracotomies (1.2%). To improve cerebral blood flow based on transcranial doppler recordings, norepinephrine was initiated in 108 cases (21.2%). In summary, the expert panel considered the course of action appropriate in 493 of 510 cases (96.7%; 95% CI, 94.7%-98.0%). Among the 17 cases (3.3%) with inappropriate course of action, 13 (76%) corresponded to a deviation from existing guidelines and 4 (24%) resulted from an erroneous interpretation of the E-FAST.

**Conclusions and Relevance:**

This prospective, multicenter cohort study found that a diagnostic resuscitation room workup for patients with blunt trauma that included E-FAST with clinical assessment and targeted chest and pelvic radiographs was associated with the determination of an appropriate course of action prior to WBCT.

## Key Points

**Question:**

What is the added value of a diagnostic workup including an extended Focused Assessment With Sonography for Trauma (E-FAST) to support decision-making in the trauma bay?

**Findings:**

In this cohort study from 6 French trauma centers, a workup combining a systematic clinical examination, targeted radiographs, and a systematic E-FAST provided critical information to appropriately guide decisions and immediate course of action. The course of action taken following this workup was considered appropriate by an expert panel in 493 of 510 cases.

**Meaning:**

These findings reinforce the use of bedside diagnostic workup in the trauma bay before whole-body computed tomography scanning.

## Teaser

This cohort study of patients with blunt trauma treated at 6 French trauma centers uses an expert panel evaluation to examine the appropriateness of trauma care decision-making following use of extended sonography assessment in clinical workups.

## Introduction

The extended Focused Assessment with Sonography for Trauma (E-FAST) scan is a cornerstone of the management of patients with trauma in the resuscitation room.^1^ The E-FAST represents an evolution of the FAST. The FAST detects free abdominal fluid and pericardial effusion. The E-FAST adds pleural and lung ultrasonograph, to detect hemothorax, pneumothorax, or hemopneumothorax, selective intubation, lung contusion, as well as pubic symphyseal widening. Incorporating transcranial Doppler (TCD) measurements of cerebral artery flow, the E-FAST provides invaluable information on cerebral hemodynamics.^2,3,4^ When used along with targeted chest and pelvic radiographs, E-FAST can provide an initial diagnostic workup to assess patients with trauma according to advanced trauma life support standards. This diagnostic bundle aims to provide a preliminary injury cartography and guides initial, sometimes lifesaving interventions before complete assessment. This initial cartography is by nature preliminary in advance of whole-body computer tomography (WBCT), which is considered criterion standard.^5,6,7,8^

A workup with clinical examination, radiographs, and E-FAST provides information to identify immediate therapeutic needs and develop a tentative plan. In time-critical circumstances, this workup may be the only information available to advise decision-making, because the CT scanner is not rapidly accessible or the clinical condition of the patient requires expedient admission to the operating room. Clinicians have relied more and more on the E-FAST workup, and the systematic use of the chest and pelvic radiographs have decreased.^9^

The diagnostic performance of the individual sections of the E-FAST, namely abdominal, thoracic, pericardial, pelvic, and transcranial, are well-established. The basic FAST provides a reasonable performance to detect intra-abdominal free fluid, pneumothorax, pericardial effusion^10,11^ and pubic symphyseal widening^12^; this performance is attenuated in hemodynamic instability.^13^ Transcranial Doppler is reliable to detect cerebral hypoperfusion.^14,15,16^ A 2019 meta-analysis^11^ ascribed to the E-FAST a rule-in, but not rule-out, capacity. Despite widespread adoption of the E-FAST,^1,9,17^ its clinical utility E-FAST remains insufficiently studied. Peytel et al^18^ performed their landmark study combining FAST and radiographs before widespread adoption of the E-FAST. To our knowledge, no prospective multicenter study has attempted to answer this question.^9^ This fact provided the rationale for the present study.

This multicenter prospective cohort study of patients with blunt trauma explored how often the course of action in patient resuscitation was appropriate based on a resuscitation room workup associating E-FAST with a systematic clinical assessment and radiographs before a WBCT.

## Methods

### Setting and Data Collection

This prospective, observational multicenter cohort study was conducted at 6 designated level I trauma centers in France. The study protocol was approved by the institutional review board of Comité Protection des Personnes Ouest. Patients or next of kin provided verbal consent according to a validated process by the institutional review board; patients could withdraw initial consent. Investigators registered the study with ClinicalTrials.org (NCT03699670). The national data protection agency (CNIL) approved the collection of anonymized patient data according to a convention signed by the Research Directorate of the Grenoble University Hospital. The manuscript followed the Strengthening the Reporting of Observational Studies in Epidemiology (STROBE) reporting guideline for prospective, observational cohort studies.

All consecutive, directly admitted patients to 1 of the participating centers were screened for inclusion. All adult patients with blunt trauma corresponding to a grade A or grade B injury using the Northern Alp Trauma Network triage criteria were included (TRENAU; eFigure in the Supplement). Patients were not included in the case of penetrating trauma, age below 18 years, no consent, patient under legal guardianship, after secondary transfer, or death in the resuscitation room.

Trained research assistants collected anonymized patient data into a standardized and secure collection tool (Voozanoo) stored on protected servers at Grenoble University. These assistants performed data management along guidelines to ensure data quality and completeness under supervision of an academic statistician (B.B.). Patient follow-up lasted 28 days.

### Clinical Management

Clinical management in the resuscitation room was at the discretion of the trauma team leader along French guidelines. A mandatory workup consisted in: (1) assessment of clinical context according to ATLS guidelines, with abnormal findings reported in patients’ medical records; (2) E-FAST by a board-certified member of the trauma team as soon as possible after arrival in the resuscitation room; (3) chest and pelvic radiographs as indicated according to the clinical assessment by the trauma team leader, with indication and any abnormality were reported in the patient’s medical record.

All clinicians performing the E-FAST were experienced board-certified trauma clinicians, either registrar or consultant level, with at least 2 years of experience in trauma management. The E-FAST is part of the formal 5-year training curriculum in anesthesia and critical care in France. The E-FAST was to be performed as soon as possible and to not exceed 15 minutes. It consisted of: standard FAST of the hepatorenal, splenorenal, Douglas, and subcostal windows to detect intrabdominal free fluid and pericardial effusion; thoracic FAST of the bilateral lung base, apical, and anterior chest windows to detect pleural effusion (hemothorax) and lung sliding; pubic FAST to detect symphyseal widening (ie, greater than 25 mm); TCD to detect low cerebral blood flow in patients with a suspicion of traumatic brain injury (Glasgow Coma Scale score below 14) with bilateral measurement of pulsatility index and diastolic blood flow velocity on the mean cerebral artery. Pulsatility index of 1.4 or blood flow velocity of 20 cm/s or lower was considered abnormal.^14^ The integral information obtained from the clinical workup (ie, clinical context, E-FAST, and radiography) along with the circumstantial information should prompt the responsible trauma team leader at his or her discretion to initiate any of the following therapeutic actions before the WBCT: abstention from any action, pelvic binder placement, resuscitative endovascular balloon occlusion of the aorta insertion, needle or thoracostomy decompression and/or chest drain, decompression of pericardial tamponade (drain or open), hemorrhage control thoracotomy, damage control laparotomy, targeted angioembolization, and cerebral blood flow improvement (ie, initiate fluid bolus, norepinephrine administration, or osmotherapy).

### Primary Outcome Criterion

All therapeutic options listed above constituted the possible global course of action, including abstention. The observed course of action was assessed by 2 independent board-certified physicians (specializing in anesthesia and critical care and echography) with more than 10 years of experience in managing patients with major blunt trauma. The experts were recruited from a college of French trauma experts; all participated in trauma guideline development. The primary outcome criterion was the level of appropriateness of the observed course of action as assessed by the expert panel by a simple binary outcome (appropriate or inappropriate). In case of a disagreement between the 2 experts, a masked third expert made the final decision.

The expert clinicians had all available clinical information at their disposal including the complete injury burden from the WBCT performed for their assessment. Deliberately, no preformatted global course of action was imposed on the experts to mimic an adaptive decision-making process comparable with a clinical setting. Experts were not aware of the study hypothesis. A script concordance based on 2 virtual standardized cases tested the agreement between experts before the study (1 case had to be considered as appropriate and the other 1 had to be considered as inappropriate); a Cohen κ quantified the level of agreement.

### Hypothesis

The initial diagnostic workup combining systematic clinical examination, E-FAST, and targeted pelvic and chest radiographs generates sufficient information to adopt an appropriate course of action before a WBCT. For each case, the integrity of all decisions and actions (including no action) were considered appropriate if in agreement with the expert panel. The global course of action was considered inappropriate if at least 1 decision or action was not in agreement. Based on the initial work by Peytel et al,^18^ the investigators expected a level of agreement above 95% between the observed course of action and assessment by the expert panel.

### Secondary Outcome Criterion

To determine the decision-making value of each diagnostic modality, after discharge of the patient from the resuscitation room, the trauma team leader in charge, and who was not aware of study hypothesis, estimated which element of the diagnostic workup affected his or her decision-making about treatment choice before the WBCT given the following choices: clinical context and examination exclusively, clinical examination and E-FAST, clinical examination and radiographs, and the combination of clinical examination, E-FAST, and radiographs. Additionally, the duration of E-FAST was categorized as less than 5 minutes, between 5 and 10 minutes, and more than 10 minutes, and all individual decisions accounted for.

### Sample Size

Based on data by Peytel et al^18^ reporting 98% of agreement in a comparable setting, we considered a 95% rate of agreement (between observed therapeutic actions and the expert) as appropriate. For this level of expected agreement and an intraclass correlation coefficient of 0.05 for 6 centers, the inclusion of 435 patients was necessary for a 95% CI and a significance level of 5%.

### Statistical Analysis

An academic statistician performed all calculations (B.B.). Descriptive statistics provided the distribution of clinical characteristics with means and standard deviations (or medians with interquartile ranges) for continuous variables and absolute numbers with percentages for categorical variables. The primary outcome criterion, the level of agreement between observed course of action and expert panel assessment, was calculated for all decisions per case on the entire cohort. The level of agreement was expressed as percentage and the confidence interval calculated according to Wald statistics (ie, bilateral confidence interval as binominal proportion). The number of individual decisions was quantified. Observed and projected mortality rates according to TRISS (Trauma Injury Severity Score) were compared with a 1-sample *Z* test comparing proportions. Our analysis did not require consideration of predictors, confounders, or effect modifiers. No subgroup or sensitivity analysis was performed. Concerning missing data, research assistants in each center retrieved all missing data. Two-sided *P* values lower than .05 were considered statistically significant. All statistical analyses were performed in February 2022 using Stata Standard Edition version 16 (StataCorp).

## Results

**Figure 1.**
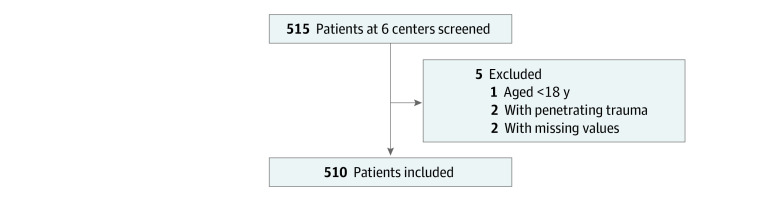
Flowchart of the Study

From November 5, 2018, to November 5, 2019, 515 patients were screened for inclusion and 510 (99.0%) fulfilled the inclusion criteria (Figure 1; eTable 1 in the Supplement). The median (IQR) age was 46 years (29-61 years) and the median ISS was 24 (17-34); 394 of the 510 patients (77.3%) were men (Table 1). Road traffic accidents (316 of 510 [62.2%]) and fall from heights (133 of 510 [26.2%]) predominated. More than 400 patients (78.4%) had an ISS above 15. Overall, 26 patients (5.1%) died within 24 hours of admission and 63 patients (12.7%) died within 28 days, with a median (IQR) TRISS of 12.4 (3.0-82.8). Observed 28-day mortality was not significantly different of the theoretical proportion given by the TRISS score (*P* = .19).

**Table 1.** Baseline Characteristics of the Cohort

Variables^a^	Patients, No. (%) (N = 510)
Sex	
Men	394 (77.3)
Women	116 (22.7)
Age, median (IQR), y	46 (29-61)
Mechanism	
Road traffic accident	316 (62.2)
Fall	133 (26.2)
Other	59 (11.6)
Systolic arterial pressure, median (IQR), mm Hg	
Prehospital	120 (100-140)
On admission	120 (100-136)
Heart rate, median (IQR), bpm	
Prehospital	90 (75-106)
On admission	87 (77-105)
Respiratory rate, median (IQR), rpm	
Prehospital	20 (16-24)
On admission	18 (15-22)
Peripheral oxygen saturation, median (IQR), %	
Prehospital	96 (89-99)
On admission	99 (96-100)
Patients with orotracheal intubation	
Prehospital	194 (38.7)
On admission	224 (44.9)
Glasgow Coma Scale, median (IQR)	
Prehospital	14 (7-15)
On admission	14 (3-15)
Patients with GCS <8	
Prehospital	155 (30.8)
On admission	185 (37.1)
Patients with a blood product transfusion first 24 h	113 (23.4)
No. of RBC in 24 h, median (IQR)	4 (2-7)
Mortality, median (IQR)	
24 h	26 (5.1)
28 d	63 (12.7)
TRISS, median (IQR)	12.4 (3.0-82.8)
ISS, median (IQR)	24 (17-34)
Patients with an ISS >15	400 (78.4)
AIS per body region, median (IQR)	
Head and neck	2 (0-4)
Face	0 (0-1)
Thorax	3 (0-4)
Abdomen and pelvis	0 (0-3)
Extremity	2 (0-3)
Surface	1 (0-1)

Abbreviations: AIS, abbreviated injury scale; GCS, Glasgow Coma Scale; ISS, injury severity score; RBC, red blood cell concentrate; TRISS, trauma injury severity score.

^a^ Missing values include: age (2 patients), mechanism (2 patients); systolic blood pressure (prehospital, 34 patients; intrahospital, 13 patients), GCS (prehospital, 7 patients; intrahospital, 11 patients), heart rate (prehospital, 39 patients; intrahospital, 22 patients), oxygen saturation (prehospital, 91 patients; intrahospital, 35 patients), respiratory rate (prehospital, 296 patients; intrahospital, 178 patients), intubation (prehospital, 8 patients; intrahospital, 11 patients) RBC transfusion (26 patients), ISS (7 patients), TRISS (27 patients), 24-h mortality (13 patients), 28-d mortality (7 patients), AIS (head and neck, 51 patients; face, 83 patients; thorax, 39 patients; abdomen and pelvis, 69 patients; extremity, 58 patients; surface, 53).

An ATLS-style physical examination was completed in all patients. An abdominal-pericardial and a thoracic ultrasonography scan E-FAST was performed in 507 patients (99.4%), a symphysis ultrasonograph in 444 cases (87.1%), and a TCD in 353 patients (69.2%) (Table 2). A chest radiograph was obtained in 300 (58.8%) and a pelvic radiograph in 284 cases (55.7%). The majority of E-FAST examinations 344 (68.4%) were performed under 5 minutes. Absence of lung sliding on thoracic ultrasonograph and pneumothorax on chest radiograph were recorded as the most frequent abnormalities.

**Table 2.** E-FAST and Radiograph Characteristics

Variable	Patients, No. (%) (N = 510)
Duration of E-FAST, min^a^	
<5	344 (68.4)
5-10	142 (28.2)
10-15	17 (3.4)
Patients with TCD	353 (69.2)
Pulsatility index >1.4	67 (18.8)
Diastolic velocity <20 cm/s	53 (15.0)
Abnormal	
PI and diastolic velocity	40 (11.2)
PI or diastolic velocity	80 (22.5)
Patients with FAST abdomen	507 (99.4)
Free abdominal fluid	69 (13.6)
Pericardial fluid	8 (1.6)
Patients with thoracic ultrasonography	507 (99.4)
Pleural fluid	51 (10.1)
No lung sliding	101 (19.8)
Patients with symphyseal ultrasonography	444 (87.1)
Symphyseal widening	24 (5.4)
Patients with chest radiograph	300 (58.8)
Pneumothorax	41 (13.6)
Diaphragmatic hernia	2 (0.7)
Pleural effusion	21 (7.0)
Patients with pelvic radiograph	284 (55.7)
Abnormal pelvic radiograph	27 (9.5)

Abbreviations: E-FAST, extended Focused Assessment With Sonography for Trauma; PI, pulsatility index; TCD, transcranial Doppler.

^a^ Value of duration was missing for 7 patients.

**Figure 2.**
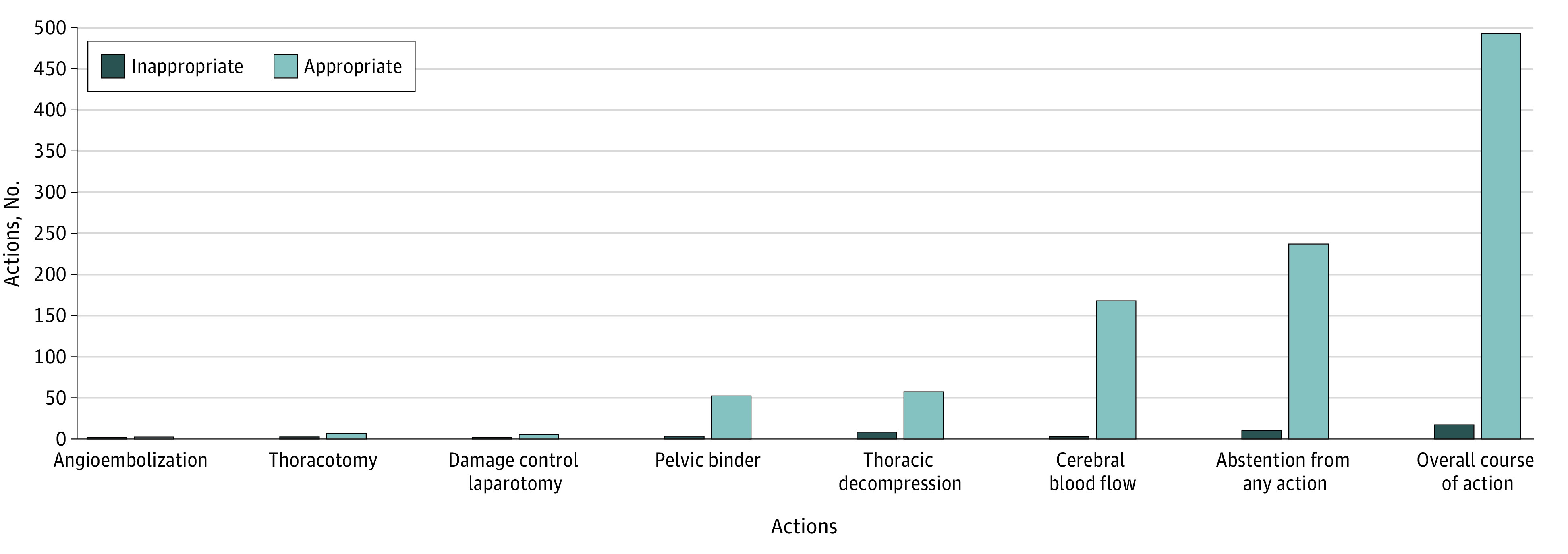
Distribution of Appropriate and Inappropriate Course of Action per Type of Action

Regarding the primary outcome criterion, all experts were fully concordant in their assessment of the 2 virtual cases (κ = 1.0). The course of action was considered appropriate by the expert panel in 493 of 510 cases (96.7%; 95% CI, 94.7%-98.0%) (Figure 2). The course of action was considered inappropriate in 17 of 510 cases (3.3%). Among these, 13 of 17 (76%) consisted in a guideline deviation and 4 (24%) resulted from an erroneous interpretation of the E-FAST findings (eTable 2 in the Supplement).

A total of 524 decisions were taken; the workup suggested therapeutic abstention in 233 decisions (44.5%) (Table 3). The most frequently performed therapeutic action was optimization of cerebral blood flow with norepinephrine infusion with or without osmotherapy in 108 (20.6%) and 60 (11.4%) cases, respectively. Overall, after the workup, 6 damage control laparotomies were initiated (1.1%), 2 pelvic angioembolizations (0.4%), 6 thoracotomies (1.1%), 57 thoracic decompressions or chest drains (11%), and 52 pelvic binders (10%). Most of the abstention from action was confirmed after a combination of clinical context and E-FAST (151 of 233 [64.8%]) (Table 3).

**Table 3.** Decisions Made According to Diagnostic Modality by the Trauma Team Leader^a^

Action taken	Decisions, No. (%)				
	Diagnostic modality				Total (N = 524)
	Clinical context and examination (n = 125)	Clinical context and E-FAST (n = 263)	Clinical context, E-FAST, and radiograph (n = 98)	Clinical context and radiograph (n = 20)	
No action performed	10 (4.3)	151 (64.8)	67 (28.7)	0	233 (44.5)^a^
Optimization CBF					
Norepinephrine	55 (50.9)	43 (39.8)	0	0	108 (20.6)^a^
Osmotherapy	25 (41.7)	33 (55.0)	0	0	60 (11.4)^a^
Thoracic decompression (needle, thoracostomy, drain)	4 (7.0)	26 (45.6)	2 (3.5)	2 (3.5)	57 (10.9)^a^
Thoracotomy	1 (16.7)	3 (50.0)	1 (16.7)	1 (16.7)	6 (1.1)
Pericardial decompression	0	0	0	0	0
Laparotomy	0	4 66.7	0	0	6 (1.1)
Pelvic binder	30 (57.7)	3 (5.8)	16 (30.8)	16 (30.8)	52 (9.9)^a^
Angioembolization	0	0	1 (50.0)	1 (50.0)	2 (0.4)

Abbreviations: CBF, cerebral blood flow; E-FAST, extended Focused Assessment With Sonography for Trauma.

^a^ Missing values included: no action performed (5 patients), norepinephrine for CBF (6 patients), osmotherapy for CBF (2 patients), thoracic decompression (3 patients), and pelvic binder (2 patients).

## Discussion

This prospective multicenter study demonstrated the utility of a resuscitation room diagnostic workup in patients with blunt trauma before WBCT to adopt an appropriate course of action. This workup associated a systematic clinical examination and targeted radiography with a systematic E-FAST. This workup provides critical information to guide decisions and therapeutic actions. This comprehensive resuscitation room workup allows frequent safe abstention from action; immediate therapeutic intervention is infrequent.

The E-FAST has achieved a level of penetration in trauma care that it seems impossible to conceive of a resuscitation room workup without it. The FAST has evolved to an E-FAST, scanning the lungs and the pelvis for injury to trigger a reduction in conventional radiography use (chest and pelvis) in the trauma room.^9^ Yet, despite extensive work exploring the diagnostic performance of the E-FAST (particularly vs conventional radiography),^10,11^ only a few studies have addressed the decision-making value of the E-FAST.

Hamada et al^9^ compared a diagnostic workup with E-FAST between stable and unstable patients with trauma in a prospective single-center study. The study’s goal was to assess whether the diagnostic resuscitation room procedure could safely forgo systematic chest and pelvic radiographs in stable patients. In a cohort of 430 cases, in 148 stable patients based on an E-FAST–only assessment, the investigators did not observe a single injury that would have required immediate therapeutic action as documented by the WBCT and clinical course. Indeed, E-FAST scanning alone detected all hemothorax requiring drainage. The protocol reduced radiation exposure and overall cost. The authors concluded that a resuscitation room assessment based almost exclusively on E-FAST was both safe and cost-efficient. Reynolds et al^19^ observed in a prospective single-center study the usefulness of point-of-care ultrasonography in a diverse range of resuscitation room pathologies (42% trauma cases). Among 986 cases, ultrasonography affected diagnostic and therapeutic treatment assessments in 27%, a change in the disposition plan in 13%, and both in 28%.

An observational study from Los Angeles in 1311 patients^20^ reported a low sensitivity of a workup combining E-FAST and chest radiographs to detect clinically significant blunt chest injuries with a significant proportion of missed injuries. The study did not assess how this workup factored into decision-making. The authors ultimately recommended the systematic use of WBCT. Finally, a retrospective French cohort of 756 patients with trauma reported a higher diagnostic performance of lung ultrasonograph over chest radiograph to detect pneumothorax and hemothorax and an acceptable sensitivity of abdominal ultrasonograph to detect intraabdominal free fluid. A therapeutic benefit for the E-FAST assessment was rare (2% to 10%) but was considered appropriate in all but 1 case.^21^

The present data confirm a low frequency of immediate therapeutic actions steered by the resuscitation room workup despite a high traumatic load (mean ISS of 24 and more than 78% of patients with an ISS above 15). The most frequent therapeutic decision was abstention in 46% (233 of 510 patients), followed by optimization of cerebral blood flow in 32% (166 of 510 patients). Pelvic binder, thoracic decompression, thoracotomy, laparotomy, and embolization occur quite infrequently, between 0.4% and 10%. Unstable patients requiring immediate, lifesaving therapeutic actions are rare. Less than 10% of all resuscitation room trauma admissions are in shock and require hemorrhage control intervention.^22^ The rates observed in our study are in agreement with previous reports.^8^ To what extent the initiated therapeutic actions translate into clinical benefit for the patient remains a challenging question.

The present study confirms the safety of a diagnostic workup combining clinical assessment, E-FAST, and focused radiography to either rule out or treat conditions that pose an immediate or potential threat. This workup provides sufficient safety to proceed to the WBCT with a low probability of unexpected threats based on the primum non nocere principle. This observation stands slightly in contrast to the meta-analysis of Netherton et al,^11^ which concluded the E-FAST to be a rule-in, but not rule-out, tool despite excellent positive and negative likelihood ratios.

This study suggests that a triangulation by all 3 diagnostic modalities provides the most complete picture, particularly in unstable patients. Integration of radiographs avoids overreliance on the E-FAST and specific pitfalls for unstable patients. For instance, a chest radiograph ruling out a selective intubation in case of ambiguous left-sided lung sliding suggests the diagnosis of anterior left pneumothorax and facilitates the decision to drain the pleura. Concordantly, in unstable patients, trauma teams should deploy systematically all 3 modalities.

The individual contribution of each modality to the clinical decision is difficult to assess. However, E-FAST provides crucial information because surgical intervention (laparotomy and thoracotomy) appears mainly driven by E-FAST results.^21^ Of note in the present study is the low rate of emergency laparotomy in comparison with Peytel et al,^18^ an observation that possibly reflects the change in practice with a higher tendency to take the patient to the computed tomography scan and in favor of interventional radiology.

### Limitations

There were several limitations to our study design and conduct. First, despite a multicenter and prospective approach, randomization seemed impossible for ethical reasons. It was not conceivable to perform the assessment without E-FAST. Furthermore, the a posteriori assessment by trained experts could be subject to intrinsic bias. Despite script concordance training and a high level of observed agreement between experts, a subjective component remains and hindsight bias cannot be excluded. Finally, no strict framework was provided for the experts to assess the appropriateness of the observed course of action; such a framework might have impeded the adaptive care crucial to trauma management.

## Conclusions

This prospective, multicenter, observational study found that a diagnostic resuscitation room workup for patients with blunt trauma that included E-FAST with clinical assessment and targeted chest and pelvic radiographs was associated with the determination of an appropriate course of action before WBCT. The combined use of all 3 diagnostic modalities appears necessary in unstable patients before WBCT. In the case of a negative workup, it appears safe to proceed with the patient to the WBCT.

## Notes

Supplement. **eFigure.** Triage Grading System From TRENAU **eTable 1.** Number of Inclusions per Center **eTable 2.** List of Inappropriate Course of Actions
