# Supplementary material for: Appropriateness of Initial Course of Action in the Management of Blunt Trauma Based on a Diagnostic Workup Including an Extended Ultrasonography Scan
Source: JAMA Netw Open. 2022 Dec 7;5(12):e2245432. doi: 10.1001/jamanetworkopen.2022.45432 (PMC9856525; doi:10.1001/jamanetworkopen.2022.45432)
Supplement: Supplement. — eFigure. Triage Grading System From TRENAU eTable 1. Number of Inclusions per Center eTable 2. List of Inappropriate Course of Actions [file jamanetwopen-e2245432-s001.pdf]

Supplemental Online Content

Planquart F, Marcaggi E, Blondonnet R, et al. Appropriateness of initial course of action in the management of blunt trauma based on a diagnostic workup including an extended ultrasonography scan. *JAMA Netw Open.* 2022;5(12):e2245432. doi:10.1001/jamanetworkopen.2022.45432

eFigure. Triage Grading System From TRENAU

eTable 1. Number of Inclusions per Center

eTable 2. List of Inappropriate Course of Actions

This supplemental material has been provided by the authors to give readers additional information about their work.

© 2022 Planquart F et al. *JAMA Network Open.*

eFigure. Triage Grading System From TRENAU

Grade A: *instable despite resuscitation*

- Systolic arterial pressure < 90 mmHg despite the use of vasopressors and/or more than 1.5 L crystalloid fluids
- SpO2 < 94% despite the use of mechanical ventilation or the use of facial mask with high-flow oxygen
- GCS ≤ 8 or motor GCS ≤ 4

Grade B: *stablilized after prehospital resuscitation or anatomic criteria*

- Systolic arterial pressure > 90 mmHg or SpO2 > 94% after initial resuscitation
- GCS 9 -13
- Suspicion of spinal cord injury
- Positive extended-FAST
- Suspicion of severe pelvic injury
- Severe limb injury (amputation or crushed limb)
- Penetrating injury to haed / neck / trunk

Grade C: *Stable with high-kinetic circumstances or medical history*

- Fall from more than 6 meters, ejected/Projected/Blasted victim;
- Death in the same vehicle
- Assessment of speed accident: vehicle deformation, no seat belt, no helmet
- Fracture of two proximal long bones (femur/humerus)
- Medical history: <5 yrs or > 65 yrs, pregnancy, coagulation disorders

© 2022 Planquart F et al. *JAMA Network Open.*

eTable 1. Number of Inclusions per Center

Name of the center	Number of inclusions
Grenoble	101
Strasbourg	84
Paris La Pitié	21
Marseille	154
Nîmes	7
Clermont Ferrand	143

© 2022 Planquart F et al. *JAMA Network Open.*

eTable 2. List of Inappropriate Course of Actions

Inappropriate course of action	Guideline deviation	eFAST misinterpretation	Details
**No chest decompression or drainage**	Yes		Suspected major pneumothorax not drained in resuscitation room
**No chest decompression or drainage**	Yes		Major pneumothorax not drained in resuscitation room
**Pelvic binder placed**	Yes		Patient hemodynamically stable, eFAST negative, no pelvic injury on WBCT
**Pelvic binder placed**	Yes		Patient hemodynamically stable, eFAST negative, no pelvic injury on WBCT
**No chest decompression or drainage**	Yes		Major pneumothorax diagnosed on eFAST and respiratory compromise not drained in resuscitation room before WBCT
**No osmotherapy**		Yes	Transcranial doppler profile misinterpreted in patient with GCS 3 and signs of cerebral herniation in WBCT
**No pelvic angioembolisation**		Yes	Positive eFAST with decision in favour of emergency laparotomy without pelvic radiography; no intrabdominal injury but pelvic injury necessitating angioembolisation in the end.
**No chest decompression or drainage**	Yes		Major pneumothorax diagnosed on eFAST and radiography with respiratory compromise not drained in resuscitation room before WBCT
**No osmotherapy**	Yes		Patient GCS 7 and transcranial doppler profile in favour of raised intracranial hypertension
**No chest decompression or drainage**	Yes		Major pneumothorax diagnosed on eFAST and radiography with hemodynamic and respiratory compromise not drained in resuscitation room before WBCT
**No thoracotomy**		Yes	eFAST interpreted in favour of emergency laparotomy instead of thoracotomy; bleeding source thoracic in the end
**No laparotomy**	Yes		Positive abdominal FAST in unstable patient, transported to WBCT
**Chest drain placement**		Yes	Chest drain placed during trauma resuscitation, eFAST misinterpreted as pleural effusion/hemothorax; no fluid on drainage
**No chest decompression or drainage**	Yes		Bilateral tension pneumothorax diagnosed on eFAST with hemodynamic and respiratory compromise not drained in resuscitation room before WBCT
**No chest decompression or drainage**	Yes		Bilateral tension pneumothorax diagnosed on eFAST with hemodynamic and respiratory compromise not drained in resuscitation room before WBCT
**Pelvic binder placed**	Yes		Patient hemodynamically stable, eFAST negative, no pelvic injury on WBCT
**No chest decompression or drainage**	Yes		Insufficient prehospital thoracostomy, major pneumothorax with hemodynamic and respiratory compromise not drained in resuscitation room before WBCT

© 2022 Planquart F et al. *JAMA Network Open.*
